# Lithium ion Speciation in Cyclic Solvents: Impact of Anion Charge Delocalization and Solvent Polarizability

**DOI:** 10.1021/acs.jpcb.3c06872

**Published:** 2024-03-28

**Authors:** Ernest O. Nachaki, Daniel G. Kuroda

**Affiliations:** Department of Chemistry, Louisiana State University, Baton Rouge, Louisiana 70803, United States

## Abstract

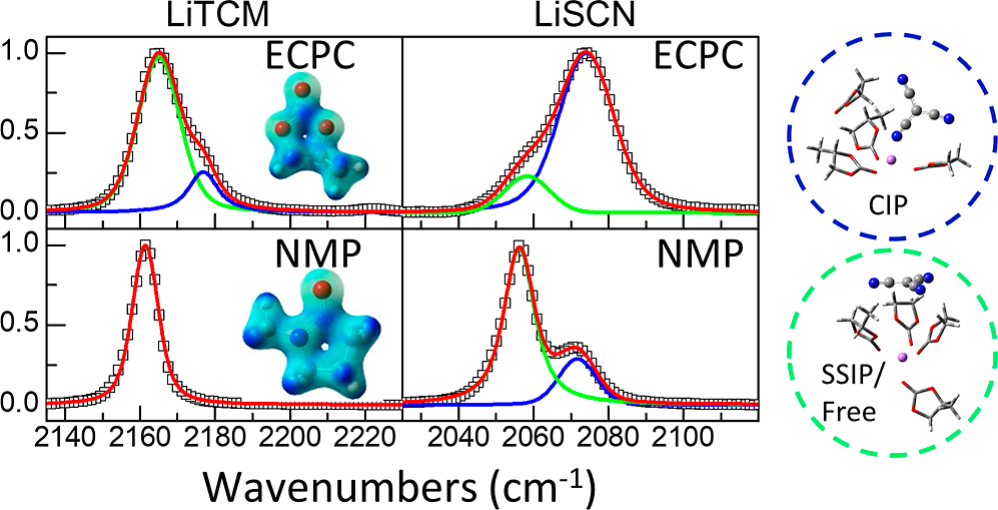

The increasing demand for lithium batteries has triggered the search for safer and more efficient electrolytes. Insights into the atomistic description of electrolytes are critical for relating microscopic and macroscopic (physicochemical) properties. Previous studies have shown that the type of lithium salt and solvent used in the electrolyte influences its performance by dictating the speciation of the ionic components in the system. Here, we investigate the molecular origins of ion association in lithium-based electrolytes as a function of anion charge delocalization and solvent chemical identity. To this end, a family of cyano-based lithium salts in organic solvents, having a cyclic structure and containing carbonyl groups, was investigated using a combination of linear infrared spectroscopy and *ab initio* computations. Our results show that the formation of contact-ion pairs (CIPs) is more favorable in organic solvents containing either ester or carbonate groups and in lithium salts with an anion having low charge delocalization than in an amide/urea solvent and an anion with large charge delocalization. Ab initio computations attribute the degree of CIP formation to the energetics of the process, which is largely influenced by the chemical nature of the lithium ion solvation shell. At the molecular level, atomic charge analysis reveals that CIP formation is directly related to the ability of the solvent molecule to rearrange its electronic density upon coordination to the lithium ion. Overall, these findings emphasize the importance of local interactions in determining the nature of ion–molecule interactions and provide a molecular framework for explaining lithium ion speciation in the design of new electrolytes.

## Introduction

Li-ion batteries (LIBs) have enabled a revolution in small electronics, from hand-held devices to robots.^1^ Electrolytes are a key component of the LIBs since they are responsible for transporting charges across the electrochemical cell.^2^ While the durability of LIBs is limited by an unstable electrolyte–electrode interface, which causes many safety issues, the LIB performance is related to the electrolyte ability to transport charges during the charge and discharge cycles.^3−6^ The development of safer and more efficient LIB electrolytes requires new insights into the molecular description of the constantly evolving electrolytes.^7^ The physicochemical properties of the electrolyte, such as the electrochemical stability and conductivity, have been linked to the chemical nature of the electrolyte components.^8−14^ In particular, the electrolyte performance is primarily dictated by the nature of the ionic speciation in the electrolyte, and the electrochemical stability is determined by the type of solvent and the anion used.^15^ Hence, the design of lithium electrolytes has been guided by selecting the appropriate solvent and anion. To this end, two important properties have been used in the selection: ionic conductivity and the ability to form a stable solid electrolyte interphase. While the latter is a very important electrolyte property, it is not simply defined by the electrolyte but by the electrode/electrolyte interphase, and so it can only be properly evaluated under operando conditions.^16−19^ On the other hand, ionic conductivity is a property that depends on the microscopic interactions of the electrolyte and can be evaluated ex situ.

The ionic conductivity of a solution is typically modeled in terms of the speciation of the electrolyte ionic components. Hence, a large number of studies have been focused on describing the ionic speciation of lithium ions in different electrolytes.^20−22^ At the microscopic level, computational and experimental studies have shown that the chemical structure of the anion strongly influences the ion associations;^23−25^ the free energy of contact-ion pair (CIP) and solvent-separated ion pair (SSIP) formation is strongly influenced by the chemical nature of the anion and the solvent;^13^ the solvation shell of the lithium ion is dictated by the structure of the solvent molecules;^26−28^ the solvent coordinates lithium ions with four molecules;^26,27,29^ and the lithium ion solvation structure determines the nature of ion speciation.^11,28,30,31^ Furthermore, the macroscopic properties of the electrolyte are strongly influenced by the chemical nature of the solvent and the counteranion in the electrolyte.^32−38^ For example, the formation of CIPs, aggregates (i.e., clusters of CIPs), and SSIP solvation species influences not only the conductivity and viscosity but also the electrochemical window of the electrolyte.^32,39−43^ Hence, understanding how the counteranion and the solvent influence the ion speciation is paramount to electrolyte design.

**Scheme 1:**
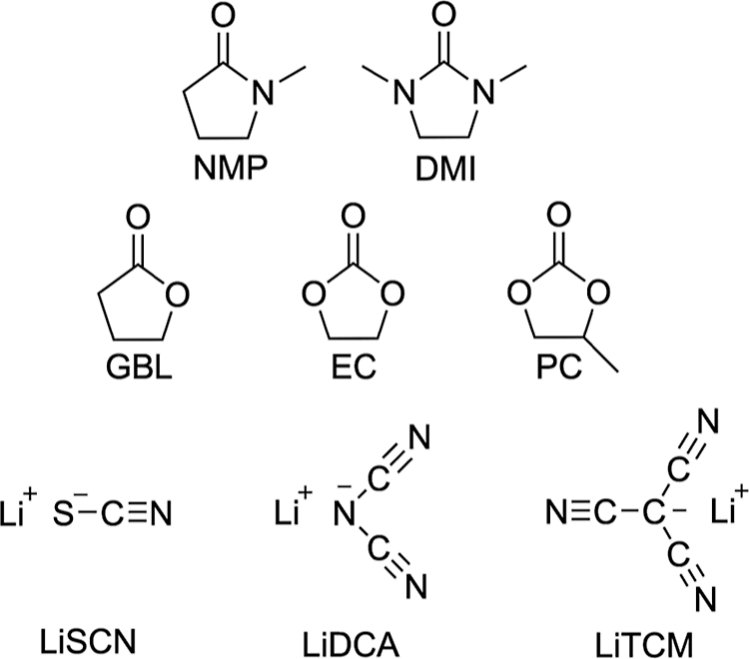
Lithium Salts (Bottom Row) and Amide-Based and Carbonate-Based Solvents (Top Two Rows)

The current study explores the effect of the molecular features of both the solvent and the anion on the ionic speciation for solvents commonly used in lithium electrolytes. For this purpose, lithium salts having anions with different degrees of symmetry and size are investigated. In addition, two different categories of solvents are studied, i.e., those containing a carbonyl group and oxygen atoms (carbonate and ester groups) and those containing a carbonyl group and nitrogen atoms (amide and urea groups). Specifically, *N*-methyl-2-pyrrolidone (NMP), 1,3-dimethyl-2-imidazolidinone (DMI), γ-butyrolactone (GBL), and a 1:1 molar ratio mixture of ethylene carbonate (EC) and propylene carbonate (PC) were selected because of their potential or current application in LIBs (Scheme 1).^44−46^ In addition, all these solvents present similar cyclic structures (Scheme 1) and high dielectric constants (larger than 30).^47,48^ The former property minimizes any possible changes in the Li^+^ solvation structure due to changes in the solvent molecular size. To systematically investigate the effect of the anion structure on the ionic speciation, a family of cyano-based lithium salts [lithium thiocyanate (LiSCN), lithium dicyanamide (LiDCA), and lithium tricyanomethanide (LiTCM)] was chosen because their nitrile stretch acts as an intrinsic infrared probe.^49−54^ Moreover, concentration-dependent Fourier transform infrared (FTIR) spectroscopy is used to unambiguously assign the speciation of the anion.^50,55−58^ Finally, complementary *ab initio*computations provide not only the energetics of lithium ion solvation and ion-pair interactions^26,59,60^ but also a window into the molecular origins of ion associations from the structural and electronic features of the ionic species.^61−63^

## Experimental and Theoretical Methods

### Sample Preparation

Lithium thiocyanate (LiSCN·*x*H_2_O, LiSCN > 63% Alfa Aesar), LiDCA (> 99% IOLITEC), and LiTCM (> 99% IOLITEC) were used. All salts were dried in a vacuum oven at 100 °C for 48 h. EC (> 99% Acros Organics) was used as received. PC (99.5% Acros Organics), GBL (≥ 99% Sigma-Aldrich), NMP (> 99% Fischer Chemicals), and DMI (98% Alfa Aesar) were dried in 4 Å molecular sieves for at least 24 h before use, which resulted in less than 100 ppm of water as determined by Karl Fischer titration. The ECPC solvent was prepared as a 1:1 molar ratio of EC and PC. The solutions contain 100 mM of lithium salt due to the small solubility of some of the salts in the solvents. All samples were prepared in a nitrogen-filled glovebox to minimize water contamination.

### Linear Infrared Spectroscopy

The linear infrared (IR) measurements of the samples were performed with a Bruker Tensor 27 FTIR spectrometer with a liquid nitrogen-cooled narrow-band mercury cadmium telluride detector. The FTIR resolution is 0.5 cm^–1^. An average of 40 scans were used for each spectrum. Samples were held between two 2 mm CaF_2_ windows separated by different Teflon spacers ranging from 25 to 100 μm. The FTIR sample cells were assembled in a nitrogen-filled glovebox.

### Density Functional Theory

**Scheme 2:**
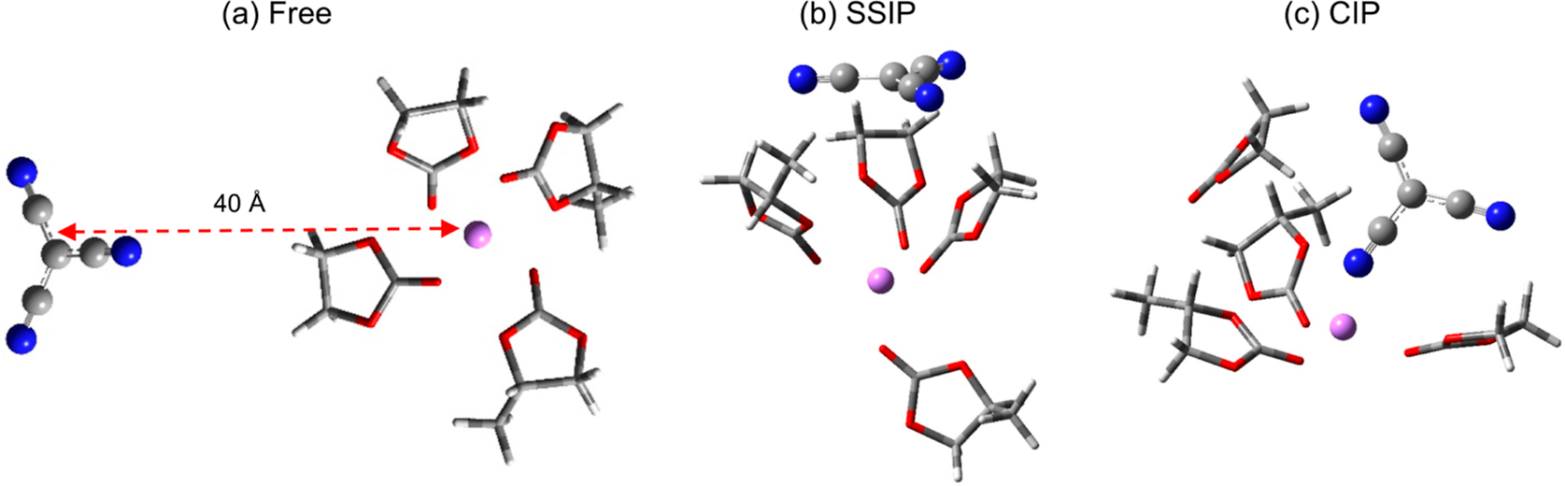
Solvation Structures for the (a) Free, (b) SSIP, and (c) CIP Configurations of LiTCM in ECPC

Ab initio calculations using the density functional theory (DFT) method were performed at the PBEPBE level of theory, using a 6-31+G (d,p) basis set,^64,65^ since it has been previously demonstrated that this combination produced results similar to those obtained from the MP2 level of theory.^66,67^ In this study, only the first solvation shell around the Li^+^ ion was explicitly considered without any implicit solvent models. The methodology was chosen based on previous work showing that the use of a dielectric continuum does not alter the energetic trends of the Li^+^–solvent interaction beyond the first solvation shell.^68,69^ The initial molecular structures, tetrahedral Li^+^ solvation shells (Scheme 2) containing the anion at close or large distances,^28,69−72^ were first built and minimized in Avogadro software using the MMFF94 force field.^73^ In the case of thiocyanate, the coordination of the counterion to the Li^+^ was through either the N or S atom, since it was observed to be solvent-dependent (see Supporting Information). Geometry optimizations, energy and frequency calculations, and natural bonding orbital (NBO) analysis were performed using the Gaussian 16 software.^74^ In particular, frequency calculations were used to confirm that the optimized geometries of the different complexes were in an energy local minimum. The enthalpy of CIP formation from the free/SSIP anion was calculated using the same procedure described in ref (60).

## Results

### Anion Structure Effect

**Figure 1:**
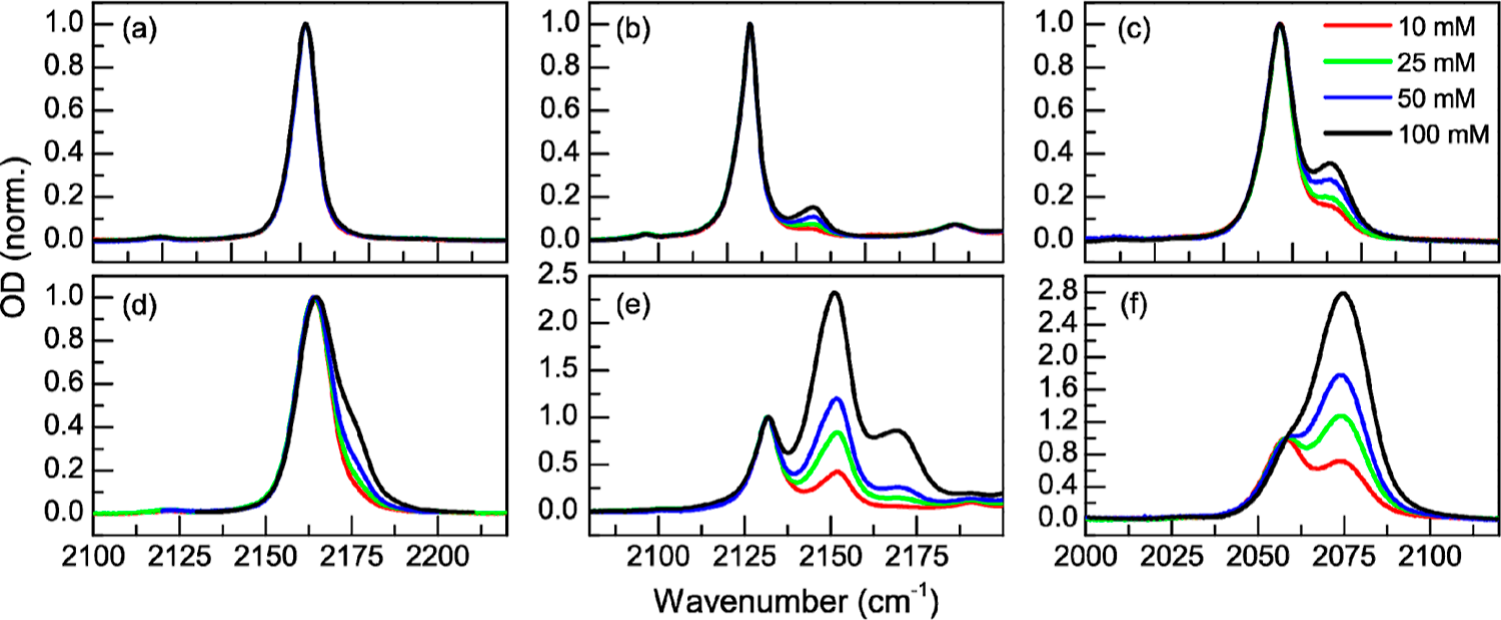
Concentration-dependent FTIR spectra of the lithium salts in different solvents. Panels (a–c) show the normalized spectra of LiTCM, LiDCA, and LiSCN in NMP, respectively, while panels (d–f) are those of ECPC. Red, green, blue, and black lines correspond to 10, 20, 50, and 100 mM, respectively. The spectra are normalized to the maxima except for panels (e,f), which are normalized to the low frequency bands at 2131 and 2058 cm^–1^, respectively.

The effect of the anion structure was investigated in two different solvents, ECPC and NMP, since their results are similar to those of GBL and DMI, as detailed in the next section. The concentration-dependent FTIR spectra in the nitrile stretch region (2000–2200 cm^–1^) for the three anions investigated: thiocyanate (SCN), dicyanamide (DCA), and tricyanomethanide (TCM) are displayed in Figure 1. The normalized FTIR spectra (Figure 1) present two or more bands in the CN stretch region for the different salt–solvent combinations, except for LiTCM in NMP where only one band is observed. Previous assignments and the FTIR of the solutions at low salt concentrations (10 mM) show that CN stretch bands of the “free” anion are located at ∼2163 cm^–1^ for the TCM ion, at ∼2128 cm^–1^ for the DCA ion, and at ∼2057 cm^–1^ for the SCN ion.^54,75^ The assignment of the CN bands for the free SCN and TCM ions is confirmed by the FTIR spectra using tetrabutylammonium thiocyanate (TBASCN) and 1-ethyl-3-methylimidazolium tricyanomethanide (EMIMTCM) in all the studied solvents (see Supporting Information). It is important to note that the so-called “free” anion band corresponds to both the free ion and the SSIP, since linear IR spectroscopy cannot distinguish the two species.^76^ The “free ion” bands correspond to the degenerate asymmetric CN stretch of TCM,^77^ the CN asymmetric stretch of DCA,^78^ and the CN stretch of SCN.^79^ Note that the DCA ion also has other bands corresponding to symmetric stretches and Fermi resonances.^78,80,81^ At higher salt concentrations (100 mM), all the anions show a second band on the high frequency side of the free nitrile stretch, except for TCM in NMP. The high frequency bands appear at ∼2176 cm^–1^ for TCM, at ∼2148 cm^–1^ for DCA, and at ∼2073 cm^–1^ for SCN. Based on the appearance and growth of the high frequency bands with salt concentration, these bands were previously assigned to CIPs.^55^

The FTIR spectra also show that for any given solvent, the structure of the anion has a major effect on the formation of CIPs since anions with more nitrile groups have smaller CIP bands or equivalent lower concentrations. Hence, the propensity of CIP formation for the three anions is described by the following trend: SCN > DCA > TCM. Note that the same trend is obtained by evaluating the percentage of CIP from the peak areas (see Supporting Information). Overall, the CIP concentration, or equivalent of its percentage (Table 1), decreases as the anion becomes more symmetric, irrespective of the solvent.

**Table 1:** Percentage of CIPs as Modeled with Voigt Profiles

solvent	percentage of CIP (%)		
	SCN	DCA	TCM
ECPC	86 ± 1	75 ± 3	24 ± 2
GBL	83 ± 1	75 ± 4	12 ± 3
NMP	22 ± 1	11 ± 1	0
DMI	17 ± 1	10 ± 1	0

### Solvent Effect

**Figure 2:**
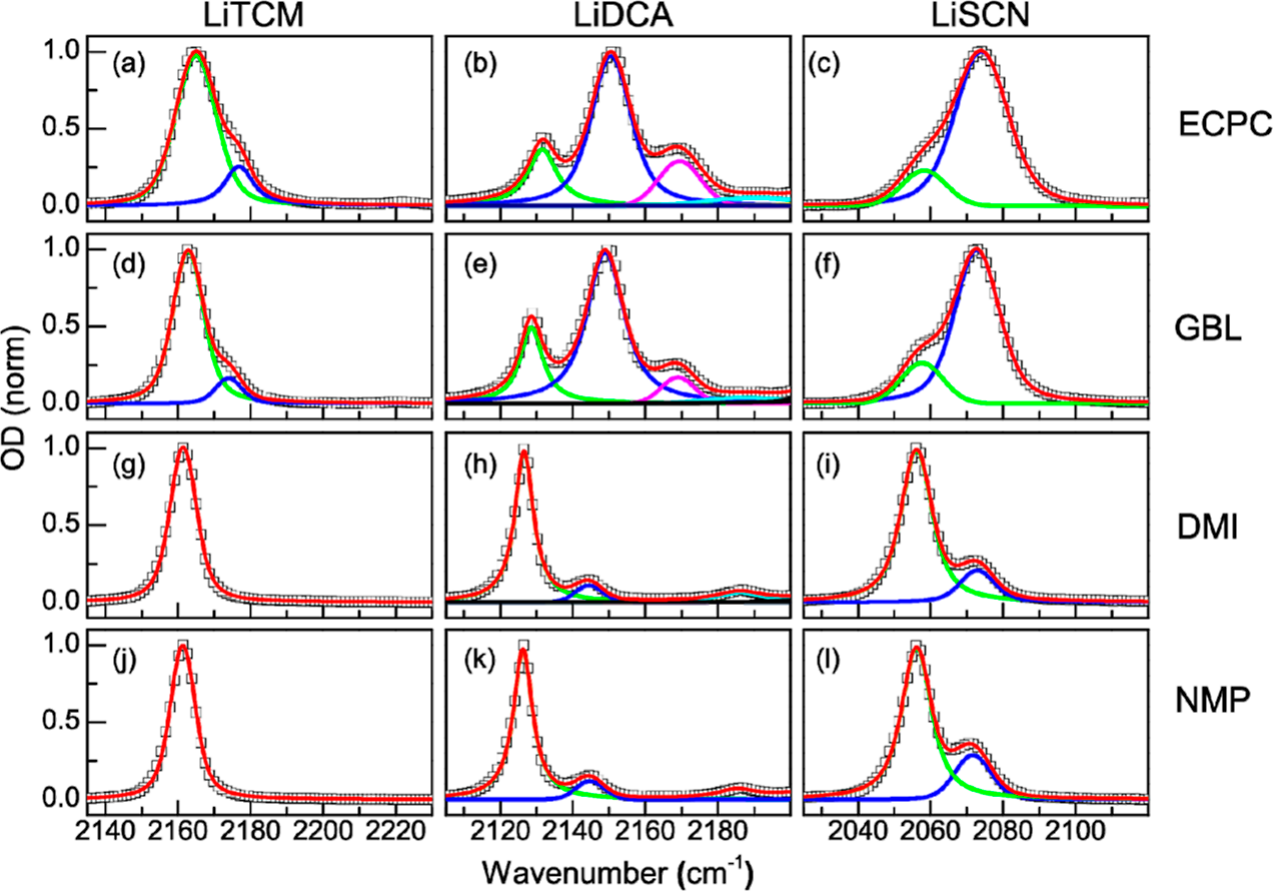
FTIR spectra of 0.1 M solutions of the different salts in the four studied solvents and their modeling. Panels (a,d,g,j) correspond to LiTCM, panels (b,e,h,k) to LiDCA, and panels (c,f,i,l) to LiSCN. The first row contains solutions in ECPC, second row in GBL, third row in DMI, and fourth row in NMP. Open squares display the experimental IR spectra, while green, blue, magenta, and red correspond to free SSIP species, CIP species, the symmetric CN stretch band of DCA, and the cumulative fitting peak, respectively.

The solvent effect, investigated in the 0.1 M solutions of the salts, reveals a significant variation in the speciation of the anions in the four investigated solvents (DMI, NMP, GBL, and ECPC) shown in (Figure 2). In particular, it is observed that there is a significant difference in the tendency to form CIPs between solvents containing only oxygen atoms (GBL and ECPC) compared and those containing both nitrogen and oxygen atoms (NMP and DMI) (Figure 2). Moreover, in the case of LiTCM, CIPs are not observed in either NMP or DMI. Surprisingly, these results also show that the dielectric constant is not a good predictor of CIP formation since NMP and DMI have lower dielectric constants than ECPC or GBL, but they form less CIPs.

## Discussion

The relative amounts of ionic species in the different solvents show that in all the studied solvents the percentage of CIP decreases with increasing symmetry of the anion (i.e., TCM > DCA > SCN) . This effect can be rationalized in terms of the anion charge delocalization. Ab initio computations show that indeed the most symmetric anion (TCM) has the most delocalized charge and vice versa (see Supporting Information). For example, SCN has ∼57% of its negative charge in its nitrile group, while TCM has ∼70% of its charge shared among its three nitrile groups.

**Figure 3:**
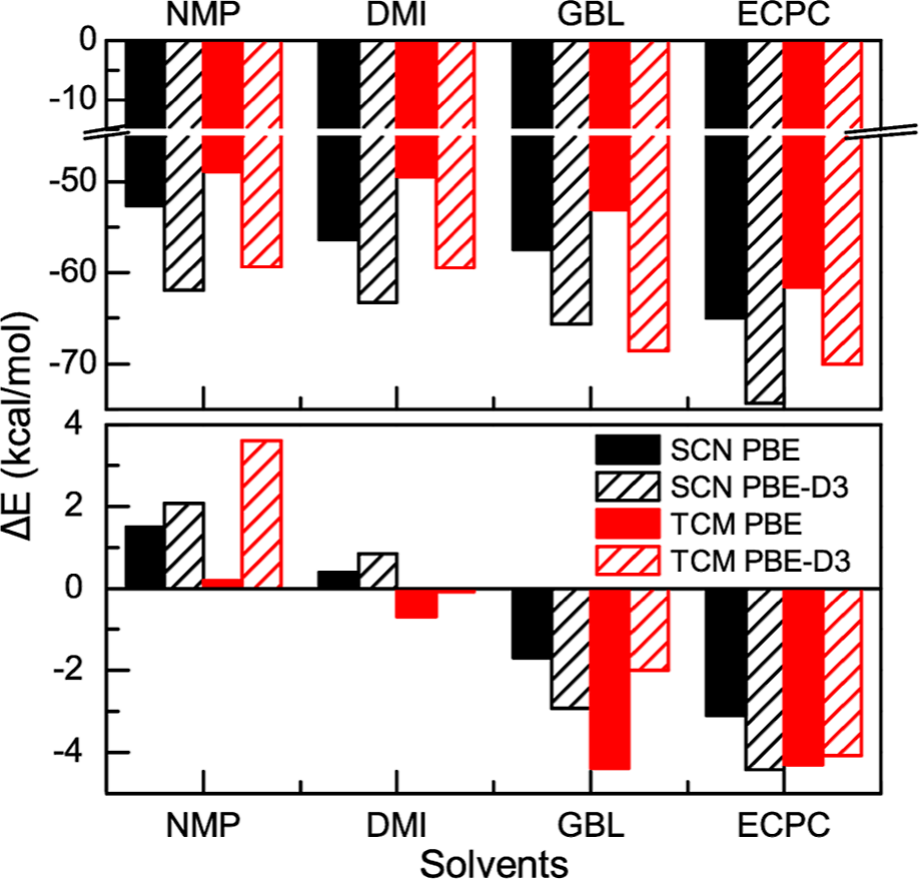
Energetics of CIP formation. Panel (a) corresponds to CIP formation energy from free ions and panel (b) from SSIPs, as described in the text, in NMP, DMI, GBL, and ECPC. The black and red solid bars correspond to the TCM and the SCN anion without the empirical dispersion corrections, while the stripped bars with the same colors correspond to the energy change when empirical dispersion correction is included.

Computations also show that SCN appears to have a larger propensity to form CIPs compared to TCM in the studied solvents when compared to the free ion (Figure 3). This trend in the SCN propensity of forming CIPs is less clear when the SSIP is used as reference. It is important to note that the energetics of the CIP formation as a function of the anion is small (<5 kcal/mol), which makes it less quantitative due to its high dependence on the level of theory, the DFT functional, and the cluster size (see Figure 3 and Supporting Information). However, one can use the charge delocalization of the individual anions predicted by DFT to explain the observed experimental results. In this context, SCN has the least delocalized charge and is more energetically favored to interact with the lithium ion (i.e., form CIPs) when compared to the two other ions due to the strong directionality of the Columbic interaction. Comparatively, TCM has the highest charge delocalization, which reduces the electrostatic interaction with Li^+^ and decreases its tendency to form CIPs. As expected from the trend of the charge delocalization, DCA is situated between the SCN and TCM, resulting in lower concentrations of CIPs than SCN but larger than that of TCM. The trend of CIP formation demonstrates the importance of the anion structure and its ability to spread its charge for the formation of CIPs.

To further investigate the effect of solvent and anion structures on the CIP formation, DFT calculations of the different species (free, SSIPs, and CIPs as shown in Scheme 2) were performed for the two extremes of anion charge delocalization (i.e., SCN and TCM). It was previously demonstrated that the arrangement of the SCN anion in the CIP solvation structure is solvent-dependent.^50,57^ Hence, to accurately represent the energetics of the process, the CIP structure involving SCN was first determined by using IR spectroscopy (see Supporting Information). The computations show that for the process of CIP formation from a free ion as given by
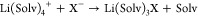


a trend as a function of the solvent is observed, where ECPC or GBL favors the CIP formation when compared to NMP or DMI. Note that this trend is not affected by the chemical identity of the anion, the use of larger solvent clusters, the inclusion of dispersion interactions, or the DFT functionals (see Supporting Information). In fact, increasing the number of solvent molecules from 4 to 6 in the Li^+^ SSIP and CIP solvation structures (see Supporting Information Figure S6) results in a change in the Δ*E* (SSIP to CIP) of ≤1.5 kcal/mol (see Supporting Information Figure S4), but the trend in the energetics as a function of solvent is preserved. Hence, it is inferred from the energetics of CIP formation (Figure 3) that the chemical identity of the Li^+^ solvation shell plays an important role in ion-pair formation. The trend in the calculated energetics as a function of the solvent is in reasonable agreement with the experimental trend (Figure 2 and Table 1), but does not explain the lack of CIP formation for TCM in either the amide- or the urea-containing solvents (i.e., DMI and NMP).

The energetics of CIP formation from free ions might not provide a full picture of the process because it does not include the effect of solvation on the anion. Moreover, it could be overestimated given that IR spectroscopy cannot distinguish between free and SSIP species.^76^ Therefore, to minimize the anion solvation effects and account for other possible ionic species, the formation of CIPs from SSIPs was also considered. As in the case of the CIP from the free anion, the DFT energetics of CIP formation starting from SSIPs (i.e., SSIP → CIP) for the different solvents (Figure 3) show that, regardless of the anion identity, the SSIP is more energetically stable in either the amide or urea solvents, while the CIP is the more energetically favored species in the ester or carbonate solvents. The observed trend energetics of CIP formation from SSIPs provides a thermodynamic framework to explain the experimental observations for the anion speciation as a function of solvent chemical structure, but they do not offer a molecular framework to rationalize the changes in speciation.

Several molecular parameters can be used to model the nature of ion–solvent interactions and the propensity for CIP formation among solvents.^40,69,82^ Among these parameters, the most relevant are the coordination ability of the solvent, the degree of charge delocalization of the lithium ion into the solvation shell, and the ability of the solvent molecule to rearrange its electronic density in the presence of an ion (i.e., electronic polarizability). Computational results show that the studied solvents have similar coordination abilities, since they all form tetrahedral solvation shells and the lithium–solvent distance, as described by the length between the oxygen atom of the carbonyl group (C=O) and the lithium center, presents only a slight variation among them (Table 2). In addition, the charges of the complex (Li(Solv)_4_^+^) derived by NBO analysis reveal that the lithium ion does not have a significantly different charge delocalization in the different solvent molecules to account for the variations observed experimentally and theoretically in the CIP formation (Table 2). However, NBO analysis shows that the electronic density in the solvent molecules is significantly altered upon coordination to the lithium ion.

**Table 2:** Average Li^+^ to O=C Bond Distance, the Change in Carbonyl Bond Length, and the Li^+^ Charge, After Solvent Coordination to Li^+^

solvent	⟨Li^+^···O_OC_⟩ dist. (Å)	⟨ΔC=O⟩ length (Å)	Li^+^ charge
ECPC	1.96	0.015	0.871
GBL	1.97	0.014	0.865
DMI	1.95	0.015	0.877
NMP	1.98	0.017	0.879

A quantitative description of the charge redistribution is obtained by comparing the net changes of the solvent molecule before and after its coordination to the lithium center. To this end, the charges of the solvent molecule were separated into two parts: the part that interacts directly with the Li^+^ center (i.e., the carbonyl group) and the rest of the molecule. It is important to note that the NBO analysis can be performed among different solvents because the total charge acquired by the four molecules coordinating the lithium ion is similar in all of thems (Table 2). Analysis of the NBO charges shows that the amide/urea solvents (DMI and NMP) experience a slightly larger charge redistribution both in the carbonyl group and in the rest of the molecule upon coordination to Li^+^. For example, DMI shows a change of −0.080e in the carbonyl part and +0.110e in the rest, while GBL experiences a change of −0.055e and +0.090e, respectively (Table 3). However, the effect of the electronic cloud redistribution is best represented by the percent change in charge (δ*q*/|*q*_free_|). In this case, the amide/urea solvent shows a change greater than 55%, while the ester/carbonate presents changes closer to 40% or less (Table 3). Moreover, the large changes in electronic density shown by DMI and NMP occur in both parts of the solvent molecule, indicating that the molecules with amide or urea groups have more polarizable electronic clouds than those containing ester or carbonate groups alone.

**Table 3:** Charge Distribution in Solvent Molecules Free and Coordinated to Li^+^

solvent	PC		GBL		DMI		NMP	
	rest	C=O	rest	C=O	rest	C=O	rest	C=O
free (*q*_free_)	–0.413	0.413	–0.216	0.216	–0.142	0.142	–0.036	0.036
coordinated	–0.319	0.351	–0.127	0.162	–0.032	0.062	0.071	–0.040
δ*q*	+0.094	–0.062	+0.090	–0.055	+0.110	–0.080	+0.107	–0.076
% δ*q*/|*q*_free_|	23	15	41	25	78	56	298	210

**Scheme 3:**
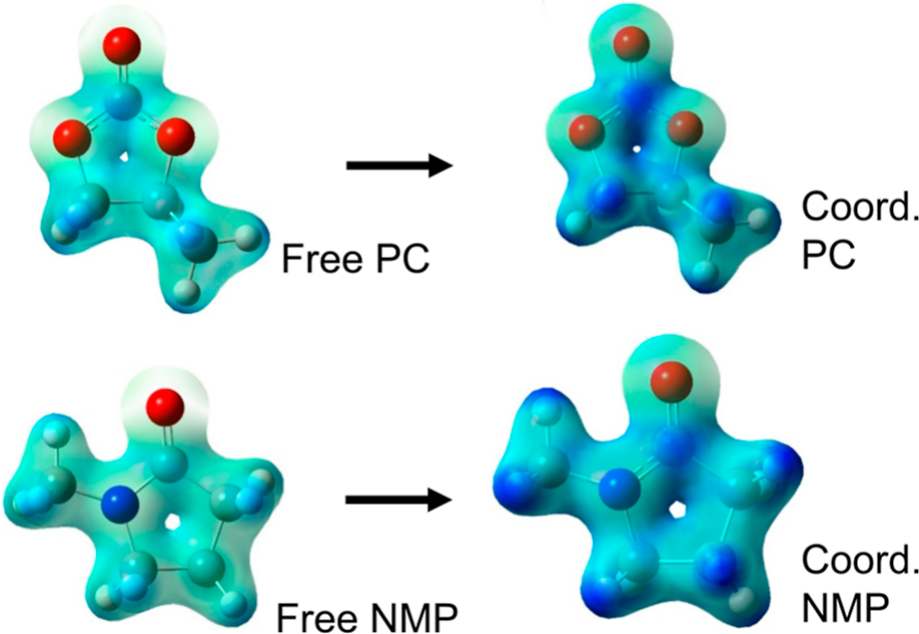
ESP Map for the Free and Coordinated PC (Top) and NMP (Bottom) Molecules; Blue Color Represents Positive Charge

The effect of charge redistribution is also observed in the electrostatic potential surface (ESP) of the free and coordinated PC and NMP molecules (Scheme 3). The ESP map shows that the NMP alkyl groups become more positively charged (blue in the scheme) compared to PC. A similar situation is observed when the ESP of GBL and DMI is analyzed (see Supporting Information). The large change in the electronic density of amide/urea-containing solvents upon coordination to lithium ions provides a molecular framework to explain the preference of SSIPs or CIPs in the different solvents. In other words, a solvent that can more effectively redistribute the positive charge in the alkyl groups should have a more suitable charge arrangement to stabilize the counteranion when it is located in its vicinity (i.e., SSIPs of Scheme 2). In contrast, solvent molecules with low to nondeformable electronic density will not transfer the Li+ charge to the surface of its first solvation shell, resulting in CIP speciation due to the lack of SSIP stabilization. The derived mechanism highlights the importance of the electronic density reorganization (polarizability) in stabilization of the different ionic species in solution.

## Summary

The present study characterizes the lithium ion solvation structure as a function of the charge delocalization of the counterion in cyclic solvents containing either amide/urea or ester/carbonate groups. Linear IR spectroscopy reveals that the degree of CIP formation decreases with increasing charge delocalization of the counterion and is more pronounced in ester/carbonate-containing solvents than in their amide/urea analogues. DFT computations attribute the CIP formation trends to the difference in the energies of the CIP formation. From a molecular perspective, the solvent electronic structure shows that the degree of electronic structure reorganization (polarizability) follows the trends of CIP formation in different solvents. Hence, the polarizability of the solvent provides a molecular framework for predicting the effect of the chemical identity of the first solvation shell on the preference of SSIPs over CIPs. Finally, the study highlights the importance of local interactions in determining ion association and energetics. The results of this study provide a framework for understanding the ion-pairing mechanisms and their effects on macroscopic properties, such as conductivity, which can be used in electrolyte design.

## Supporting Information Available

FTIR spectra of TBASCN and EMIMTCM in the studied solvents used for the assignment of the free species peaks; method of estimation of the CIP and free species including the peak areas; ESP maps for GBL and DMI; CN stretch transition dipole magnitudes; NBO charges showing the charge delocalization in the anions used; and equations of CIP formation from free and SSIP species (PDF)
