# Supplementary material for: Lithium ion Speciation in Cyclic Solvents: Impact of Anion Charge Delocalization and Solvent Polarizability
Source: J Phys Chem B. 2024 Mar 28;128(14):3408–15. doi: 10.1021/acs.jpcb.3c06872 (PMC11017243; doi:10.1021/acs.jpcb.3c06872)
Supplement: Supplementary file 1 — jp3c06872_si_001.pdf [file jp3c06872_si_001.pdf]

Supplementary Information

Lithium Ion Speciation in Cyclic Solvents: Impact of Anion Charge Delocalization and Solvent Polarizability

*Ernest O. Nachaki and Daniel G. Kuroda**

Department of Chemistry, Louisiana State University, Baton Rouge, Louisiana 70803, United States

*Address correspondence to dkuroda@lsu.edu

Assignment of the Free/SSIP FTIR peaks

The location of the free/SSIP peaks was assigned using the FTIR spectra of the salts consisting of bulky cations with high charge delocalization which minimize electrostatic interactions with the anions in the studied solvents, i.e., TBASCN and EMIMTCM (Figure S1).

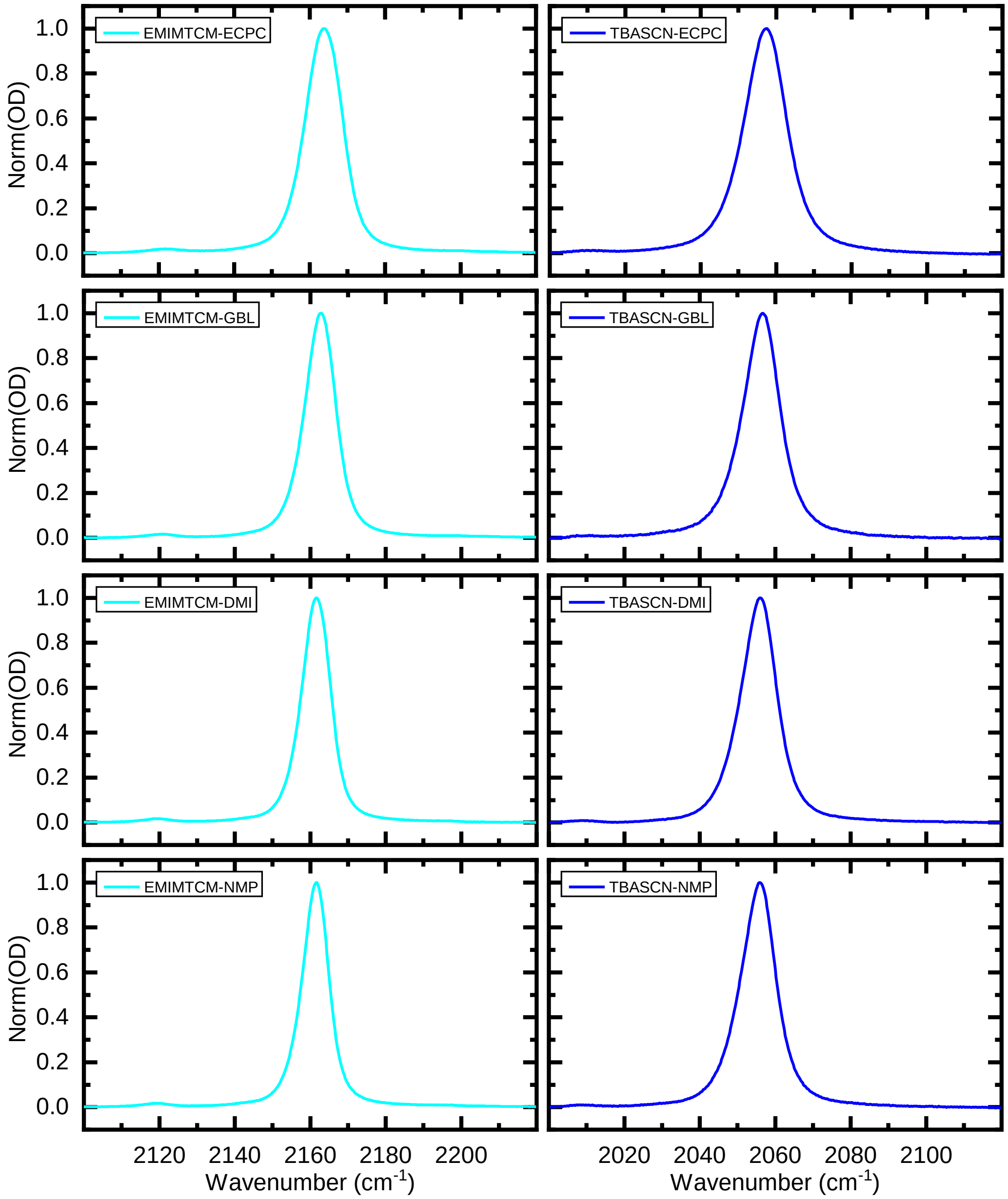

Figure S1. FTIR Spectra of 0.1 M EMIMTCM (cyan) and TBASCN (blue) in different solvents

Estimation of CIPs and Free Species

The percentage of CIP was obtained by modelling the FTIR spectra of the 0.1 M solutions with Voigt profiles, to obtain the percentage of each species from the fitted peak areas.[1-2] The peak area of an IR spectrum is primarily determined by the populations of the oscillators and magnitude of the transition dipole moment of the transition, hence population estimates are reliable in the limit of transition dipole magnitudes being similar in the different environments.[3] DFT computations show that the magnitude of the transition dipole of the CN stretch for the SSIP and the coordinated (CIP) species is similar within <20% on average (see Table S2). The estimated peak areas for the Free/SSIP and the CIP species is shown in Table S1.

Table S1. Peak areas modeled with Voigt profiles from FTIR peaks of Free/SSIP and CIP

	Free Peak Area			CIP Peak Area			Percentage CIP		
	LiSCN	LiDCA	LiTCM	LiSCN	LiDCA	LiTCM	LiSCN	LiDCA	LiTCM
ECPC	3.5±0.1	5.6±0.2	14.0±0.3	21.2±0.2	17.1±0.6	4.4±0.3	86±1	75±3	24±2
GBL	3.8±0.1	5.9±0.3	13.0±0.4	18.4±0.1	17.9±0.9	1.7±0.4	83±1	75±4	12±3
NMP	14.0±0.1	9.9±0.1	11.0±0.1	3.9±0.1	1.2±0.1	0.0	22±1	11±1	0.0
DMF	16.3±0.1	12.4±0.1	11.6±0.1	3.2±0.1	1.6±0.1	0.0	16±1	11±1	0.0

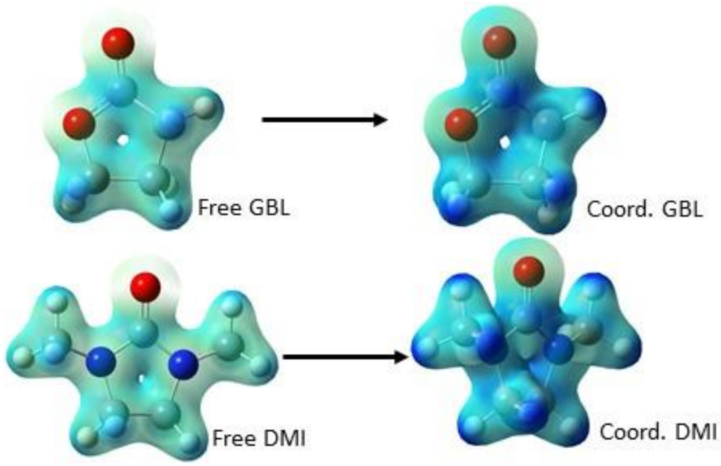

Scheme S1. Electrostatic Potential Surface Map for the Free to the Coordinated GBL and DMI.

Table S2. CN stretch transition dipole magnitude of the SSIP and CIP

	νC≡N(SCN) D(10^{-40} esu^2 cm^2)				$ν_{as}$C≡N(TCM) D(10^{-40} esu^2 cm^2)			
	SSIP		CIP		SSIP		CIP	
	cm^{-1}	D	cm^{-1}	D	cm^{-1}	D	cm^{-1}	D
DMI	2052	634	2073	483	2169	610	2174	701
					2173	655	2184	692
NMP	2049	653	2078	513	2069	631	2174	852
					2073	616	2187	723
GBL	2055	617	2079	524	2171	618	2167	739
					2172	616	2189	607
EC2PC	2050	764	2079	888	2170	698	2174	717
							2189	693
ECPC2			2073	965	2173	684	2185	821
							2186	831

1 esu^2 cm^2 = 2.5066 km/mol

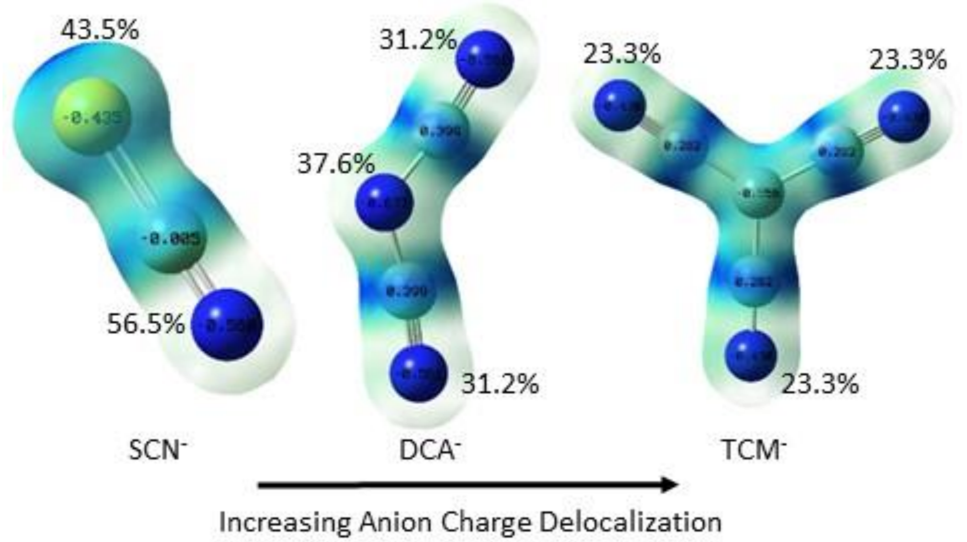

Scheme S2. NBO charges showing the percent of negative charge delocalization for SCN-, DCA- and TCM- anions.

Calculation of the energy change for CIP formation

$$\underset{\textbf{SSIP}}{\mathrm{Li(Solvent)_4Anion}} \rightarrow \underset{\textbf{CIP}}{\mathrm{Li(Solvent_3Anion)Solvent}} \quad (1)$$

The energy change of CIP formation starting with the solvent separated ion pair (SSIP) and also starting with the free anion (at 40 Å from the tetrahedral Li^+-solvent tetrahedral structure) was calculated as previously described in literature.[4] The expressions for the CIP formation are shown in Equation 1.

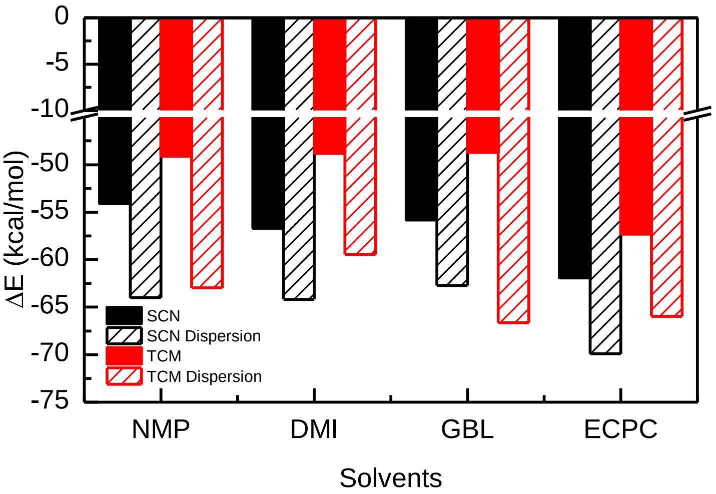

Figure S2. Energetics of SSIP formation from free species configuration for NMP, DMI, GBL, and ECPC. The black and red solid bars correspond to the SCN and TCM anions respectively without dispersion correction, while the black and red stripped lines correspond to the SCN and TCM anions respectively with dispersion correction included.

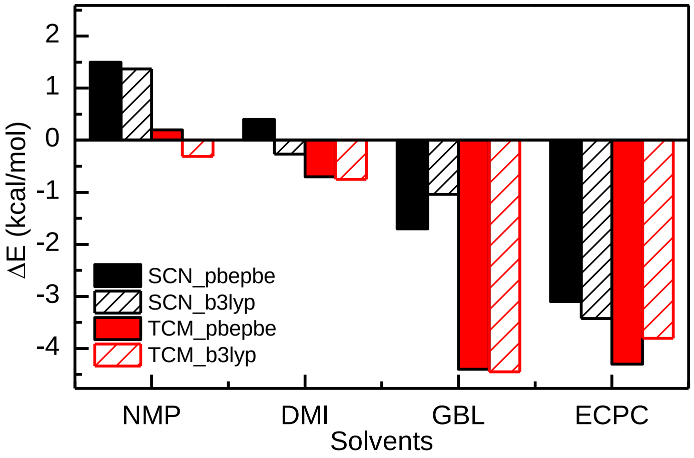

Figure S3. Energetics of CIP formation from SSIP species for NMP, DMI, GBL, and ECPC. The black and red solid bars correspond to the SCN and the TCM anion, respectivel,y using the

PBEPBE functional, while the stripped bars with the same colors correspond to the energy change when B3LYP functional is used.

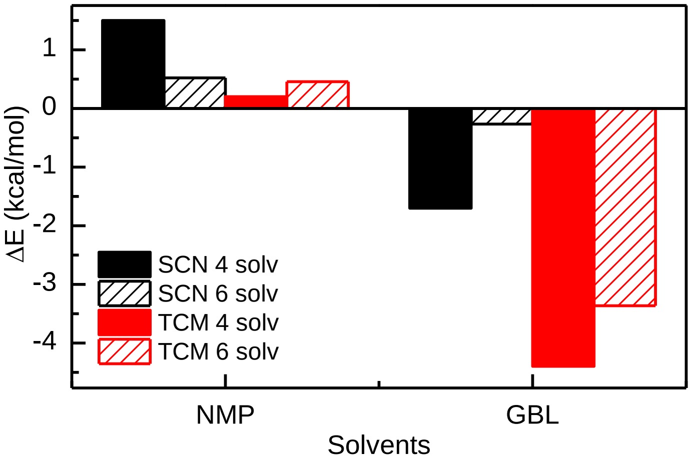

Figure S4. Energetics of CIP formation from SSIP species for NMP and GBL. The black and red solid bars correspond to the SCN and the TCM anion respectively when 4 solvent molecules are used, while the stripped bars with the same colors correspond to the energy change when 6 solvents are used.

Determination of the SCN configuration in the CIP

Previous studies have shown that the SCN CIP configuration (i.e. N-bound or S-bound to the lithium ion) is solvent dependent.[5-6] Ab-initio calculations reveal that the SC stretch of the N-bound CIP has a higher frequency, at approximately 800 cm^{-1}, compared to the S-bound CIP, which is located at approximately 740 cm^{-1}, while the free anion has an SC frequency of about 733 cm^{-1} (Table S3).

Table S3. The DFT calculated SC stretch infrared frequencies of SCN^- for the free anion, S-bound CIP (S-CIP), and N-bound CIP (N-CIP) in the studied solvents.

Solvent	ν(SC) frequency (cm^{-1})		
	Free	S-CIP	N-CIP
ECPC	733	746	803
GBL	733	738	802
DMI	733	738	800
NMP	733	736	793

Therefore, the experimental ATR spectra in the SC stretch region (700 – 950 cm^{-1}) used to establish the appropriate CIP configuration of the SCN ion in each solvent (Figure S5). The ATR spectra (Figure S5) reveals a high frequency CIP peak (approx. 900 cm^{-1}) for the ECPC solvent that is not present in the other studied solvents, demonstrating the presence N-bound CIP for ECPC and its absence in GBL, NMP and DMI.

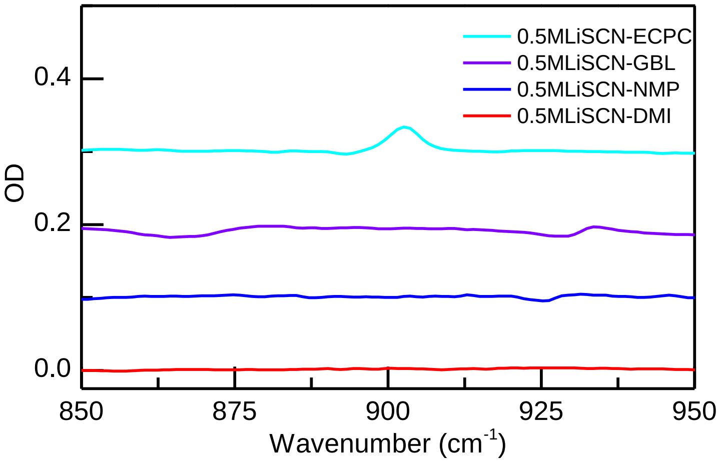

Figure S5. ATR spectra of 0.5M LiSCN in ECPC, GBL, NMP and DMI (solvent subtracted).

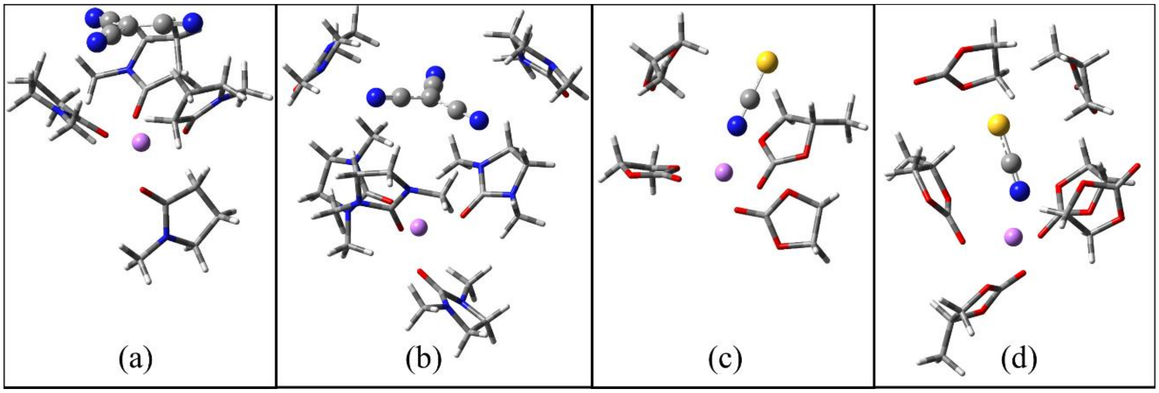

Figure S6. DFT optimized solvation structures. Panel (a) and (b) SSIP of LiTCM in 4 and 6 solvent molecules of DMI respectively, panel (c) and (d) CIP of LiSCN in 4 and 6 solvent molecules of ECPC respectively.

References

1. Fulfer, K. D.; Kuroda, D. G., Solvation structure and dynamics of the lithium ion in organic carbonate-based electrolytes: A time-dependent infrared spectroscopy study. *J. Phys. Chem. C* **2016,** *120* (42), 24011-24022.

2. Fulfer, K.; Kuroda, D., A comparison of the solvation structure and dynamics of the lithium ion in linear organic carbonates with different alkyl chain lengths. *Phys. Chem. Chem. Phys.* **2017,** *19* (36), 25140-25150.
3. Wahlers, J.; Fulfer, K. D.; Harding, D. P.; Kuroda, D. G.; Kumar, R.; Jorn, R., Solvation structure and concentration in glyme-based sodium electrolytes: A combined spectroscopic and computational study. *J. Phys. Chem. C* **2016,** *120* (32), 17949-17959.
4. Rushing, J. C.; Leonik, F. M.; Kuroda, D. G., Effect of solvation shell structure and composition on ion pair formation: The case study of litdi in organic carbonates. *J. Phys. Chem. C* **2019,** *123* (41), 25102-25112.
5. Chen, X.; Kuroda, D. G., Molecular motions of acetonitrile molecules in the solvation shell of lithium ions. *J. Chem. Phys.* **2020,** *153* (16), 164502.
6. Lee, K. K.; Park, K. H.; Kwon, D.; Choi, J. H.; Son, H.; Park, S.; Cho, M., Ion-pairing dynamics of li+ and scn- in dimethylformamide solution: Chemical exchange two-dimensional infrared spectroscopy. *J. Chem. Phys.* **2011,** *134* (6), 064506.
